# Dopamine in the auditory brainstem and midbrain: co-localization with amino acid neurotransmitters and gene expression following cochlear trauma

**DOI:** 10.3389/fnana.2015.00088

**Published:** 2015-07-22

**Authors:** Bozena E. Fyk-Kolodziej, Takashi Shimano, Dana Gafoor, Najab Mirza, Ronald D. Griffith, Tzy-Wen Gong, Avril Genene Holt

**Affiliations:** ^1^Molecular Anatomy of Auditory-related Central Systems, Department of Anatomy and Cell Biology, Wayne State University School of Medicine, DetroitMI, USA; ^2^Department of Otolaryngology, Kansai Medical UniversityOsaka, Japan; ^3^Kresge Hearing Research Institute, University of Michigan School of Medicine, Ann ArborMI, USA

**Keywords:** cochlear ablation, tyrosine hydroxylase, cochlear nucleus, inferior colliculus, Sprague–Dawley rat, hearing loss, real time PCR, immunocytochemistry

## Abstract

Dopamine (DA) modulates the effects of amino acid neurotransmitters (AANs), including GABA and glutamate, in motor, visual, olfactory, and reward systems (Hnasko et al., 2010; Stuber et al., 2010; Hnasko and Edwards, 2012). The results suggest that DA may play a similar modulatory role in the auditory pathways. Previous studies have shown that deafness results in decreased GABA release, changes in excitatory neurotransmitter levels, and increased spontaneous neuronal activity within brainstem regions related to auditory function. Modulation of the expression and localization of tyrosine hydroxylase (TH; the rate limiting enzyme in the production of DA) in the IC following cochlear trauma has been previously reported (Tong et al., 2005). In the current study the possibility of co-localization of TH with AANs was examined. Changes in the gene expression of TH were compared with changes in the gene expression of markers for AANs in the cochlear nucleus (CN) and inferior colliculus (IC) to determine whether those deafness related changes occur concurrently. The results indicate that bilateral cochlear ablation significantly reduced TH gene expression in the CN after 2 months while in the IC the reduction in TH was observed at both 3 days and 2 months following ablation. Furthermore, in the CN, glycine transporter 2 (GLYT2) and the GABA transporter (GABAtp) were also significantly reduced only after 2 months. However, in the IC, DA receptor 1 (DRDA1), vesicular glutamate transporters 2 and 3 (VGLUT2, VGLUT3), GABAtp and GAD67 were reduced in expression both at the 3 days and 2 months time points. A close relationship between the distribution of TH and several of the AANs was determined in both the CN and the IC. In addition, GLYT2 and VGLUT3 each co-localized with TH within IC somata and dendrites. Therefore, the results of the current study suggest that DA is spatially well positioned to influence the effects of AANs on auditory neurons.

## Introduction

Dopamine (DA), a monoamine neurotransmitter, has indirect effects on synaptic transmission by modulating neural circuits that contain classical neurotransmitters such as GABA, glycine, and glutamate. One way these neuromodulatory effects can be achieved is through co-release of DA and other neurotransmitters from a single neuron. Dopaminergic neurons have been reported throughout the hypothalamus, midbrain, and brainstem including the inferior colliculus (IC; Holt et al., 2005; Tong et al., 2005). In the peripheral auditory system DA has been reported to play a neuroprotective role. Specifically, lateral olivocochlear (LOC) neurons have been implicated in the protection of inner hair cells from excitotoxic damage (Puel et al., 2002; Darrow et al., 2006a,b, 2007). Immunoreactivity for tyrosine hydroxylase (TH), the rate limiting enzyme in DA biosynthesis, is decreased in LOC neurons following noise induced deafness while noise pre-conditioning seems to prevent this change (Canlon et al., 1988; Niu and Canlon, 2002; Niu et al., 2004a,b).

In the central auditory system, little is known about the role of DA in normal hearing or the effects of DA on plastic changes observed following trauma. Expression for TH is decreased in the IC for up to 3 months following cochlear ablation (Holt et al., 2005). Depending upon the IC subdivision examined, deafness related decreases in TH production (not dopamine beta hydroxylase (DBH) or phenylethanolamine-*N*-methyl transferase (PNMT)) can be either transient (days to weeks) or long lasting, i.e., months (Tong et al., 2005).

In addition to DA, neurons throughout the central auditory pathways are also known to produce GABA, glutamate and glycine. Similar to changes in TH levels, these amino acid neurotransmitters (AANs) are modulated as a consequence of cochlear trauma (Bledsoe et al., 1995; Milbrandt et al., 2000; Suneja et al., 2000; Vale and Sanes, 2002; Buras et al., 2006; Altschuler et al., 2008; Dong et al., 2010; Fyk-Kolodziej et al., 2011; Wang et al., 2011) implicating different effects on ascending versus descending and auditory versus non-auditory pathways. For example, in the IC, glutamic acid decarboxylase (GAD), the rate-limiting enzyme for GABA production, is decreased following deafness and deafness related conditions. In the cochlear nucleus (CN) the primary inhibitory neurotransmitter is glycine and deafness results in significantly reduced glycine levels (Asako et al., 2005). Expression and localization of vesicular glutamate transporters, VGLUT1-3, also change in the CN in response to deafness (Fyk-Kolodziej et al., 2011). In the superior olivary complex following deafness glycine (Buras et al., 2006) is also reduced. Since DA in several neuronal systems can be co-localized with various transmitters (Schimchowitsch et al., 1991; Gonzalez-Hernandez et al., 2001; Zhou et al., 2005; Darrow et al., 2006b; Maher and Westbrook, 2008; Hnasko et al., 2010; Stuber et al., 2010; Tecuapetla et al., 2010; Hirasawa et al., 2012; Hnasko and Edwards, 2012; Stensrud et al., 2014), this raises the question of whether DA in the auditory system is co-localized with AANs in the IC and/or the CN and whether the time course and direction of changes in DA and AAN levels correlate following deafness. In the current study, deafness related gene expression levels and co-localization of TH with markers for GABA, glycine, and glutamate are examined in the CN and IC to determine likely AAN candidates for the neuromodulatory actions of DA.

## Materials and Methods

### Animals

Specific pathogen-free adult male Sprague–Dawley Rats (Charles River, Wilmington, MA, USA) were used in accordance with the Institutional Animal Care and Use Committee (IACUC) at Wayne State University School of Medicine and conformed to guidelines issued by the National Institutes of Health and the Society for Neuroscience.

### Hearing Assessment

Hearing was assessed by ABR measures at 4, 12, and 20 kHz at the beginning of the study and those with hearing in the normal range (15–35 dB across frequencies) were included in the study. A second measure of hearing was done just prior to perfusion for animals in the deafness groups. At least a 65 dB shift from the normal control threshold averaged across the frequencies was considered necessary for continued inclusion in the study. Once animals were anesthetized (xylazine -8 mg/kg and ketamine -75 mg/kg) and placed in a sound proof booth (Hamilton–Kinder, Poway, CA, USA), three frequencies (4, 12, and 20 kHz) were tested using a speaker placed directly into the ear canal (Beyer sound source and Pyramid PA 600x stereo power amplifier). The active electrode (on top of the head), reference needle (below the recording ear) and ground (the contralateral ear) were properly placed. The sound stimulus consisted of a 15-ms tone burst, with a rise–fall time of 1 ms. Waveforms were visualized and recorded using the Data AcQusition and Real-Time Analysis (Daqarta 4.0) software package (Interstellar Research, Ann Arbor, MI, USA). The sound stimulus was started at 80 dB SPL and, in the hearing group, was decreased in 10 dB SPL steps until near threshold, then in 5 dB SPL increments until threshold could be determined. For each level, 1024 responses were averaged.

### Deafening Procedure

Each rat in the deafened group was anesthetized with, i.m., injections of xylazine (8 mg/kg) and ketamine (75 mg/kg). Local injections of 1% Lidocaine–HCl solution were made at the site of each surgical incision. Surgical procedures were performed under aseptic conditions. The skin incision was made through the post auricular region of the lateral neck. The bulla was exposed, opened and the organ of Corti was destroyed with a dental pick. The bulla was then sealed with dental cement (Durelon, Etgen, Germany) and the skin incision was closed with sutures. Following surgery, animals were injected with sterile 0.9% sodium chloride solution (1–3 ml s.c.) and allowed to recover on a heating pad.

### Real Time RT-PCR (qRT-PCR)

Under sodium pentobarbital anesthesia (90 mg/kg, i.p.), the CN and IC were rapidly dissected from 24 normal hearing rats, 12 rats at 3 days post-deafening and 12 rats at 2 months post deafening and placed in RNA Later (Applied Biosystems, Foster City, CA, USA). For each group the 12 rats were divided into four pools containing three rats each with age matched normal hearing controls for each deafened group. The RNA was then isolated as described in Holt et al. (2005). Briefly, RNA extraction was achieved by Trizol using Phase Lock (heavy) Gel tubes (Eppendorf). The concentration of RNA was quantified using A260 UV spectrophotometry and the quality of the RNA was verified by Agilent analysis (Applied Biosystem, Foster City, CA, USA). For each RNA sample 2 μg were used for cDNA synthesis (High Capacity cDNA Archive Kit). Real time RT-PCR was performed with a Mastercycler^®^ep Realplex (Eppendorf) using TaqMan Gene Expression Assays (Applied Biosystems, Foster City, CA, USA). In this study the Ct was established automatically using the Realplex software.

Thus, expression for: TH, vesicular glutamate transporter 1 (VGLUT1), vesicular glutamate transporter 2 (VGLUT2), vesicular glutamate transporter 3 (VGLUT3), GABA transporter protein (GABAtp), GABA vesicular transporter1 (VGAT), glutamic acid decarboxylase2 (GAD 65), glutamic acid decarboxylase1 (GAD 67), glycine transporter1 (GLYT1), glycine transporter2 (GLYT2), and GABA A receptor beta1 (GABRB1) was examined across groups with hypoxanthine phosphoribosyltransferase 1 (HPRT1), a housekeeping gene, used for qRT-PCR normalization. Each sample was run using the conditions described in Fyk-Kolodziej et al. (2011). Amplification efficiency was tested and calculated [efficiency (%) = 10^-1/slope^ – 1] for each assay in each brain region. Since the amplification efficiency was similar between the housekeeping gene and the genes of interest, the 2^-(ΔΔct)^ method and a two tailed *t*-test were used to quantify differential expression and determine significance (*p* ≤ 0.05). SD range was determined and applied as outlined in the technical note released by applied biosystems (www3.appliedbiosystems.com/cms/groups/mcb_support/documents/generaldocuments/cms_042380.pdf) resulting in non- symmetrical error bars.

### Immunocytochemistry

Each antiserum has been characterized in previous studies (**Table 1**). To determine the normal distribution and co-localization of TH with VGLUT1, VGLUT3, GAD67, and GLYT2 in the CN and the IC, normal hearing (*n* = 8) and deafened (*n* = 9; bilateral cochlear ablation) specific pathogen-free male Sprague–Dawley Rats were separated into three groups corresponding to time following hearing loss (no surgery-normal hearing, 3 days after surgery-3 day deaf, 2 months after surgery-2 month deaf). Animals were perfused transcardially with 4% paraformaldehyde. Once removed, the brains were post-fixed for an hour in the same solution and cryo-protected in 20% sucrose for 24 h at 4°C. Frozen brains were cut into 40 μm sections using a freezing sliding microtome and collected serially into 12 vials. Sections containing the CN and IC were incubated for 48 h at 4°C with the primary antibody (TH rabbit polyclonal antibody 1:1500–1:2000, GAD67 mouse monoclonal antibody 1:1000, GLYT2 guinea pig polyclonal antibody 1:1000, VGLUT1 guinea pig polyclonal antibody 1:1000, VGLUT3 guinea pig polyclonal antibody 1:1000 (Chemicon, Temecula, CA, USA), VGLUT3 mouse monoclonal antibody 1:100 (NeuroMab, Davis, CA, USA), DBH mouse monoclonal antibody 1:400, and PNMT mouse monoclonal antibody 1:2000 (Chemicon, Temecula, CA, USA). Following three rinses sections were incubated for 2 h with the appropriate fluorescent secondary antibody (either donkey anti-rabbit FITC, goat anti-guinea pig Texas Red, or rabbit anti-mouse Texas Red 1:500; Jackson ImmunoResearch Laboratories).

**Table 1:** Antibodies.

Antiserum/Immunogen	Species	Source/Cat. No.	Dilution	Reference
Tyrosine hydroxylase (TH) from rat pheochromocytoma	Rabbit	Chemicon, Temecula, CA AB152, USA	1:1500–1:2000	Trigueiros-Cunha et al. (2003)
VGLUT1 Amino acids 541–560 of rat VGLUT1	Guinea pig	Chemicon, Temecula, CA AB5905, USA	1:1000	D’Sa et al. (2007), Brooke et al. (2010), Fyk-Kolodziej et al. (2011)
VGLUT3 Synthetic peptide from rat VGLUT3	Guinea pig	Chemicon, Temecula, CA AB55421, USA	1:1000	Seal et al. (2008), Henny et al. (2010), Fyk-Kolodziej et al. (2011)
VGLUT3 Amino acids 546–588 of rat VGLUT3	Mouse/Clone N34/34	NeuroMab, Davis, CA 75-073	1:100	Fyk-Kolodziej et al. (2011)
Glutamic acid decarboxylase1 (GAD67) Recombinant GAD67 protein	Mouse	Chemicon, Temecula, CA MAB5406, USA	1:1000	Burianova et al. (2009), Brooke et al. (2010)
Glycine transporter 2 Synthetic peptide from rat C-terminus GLYT2	Guinea pig	Chemicon, Temecula, CA AB1773, USA	1:1000	Caminos et al. (2007), Brooke et al. (2010)
PNMT Bovine adrenal phenyl ethanolamine-*N*-methyltransferase	Sheep	Chemicon, Temecula, CA AB146, USA	1:2000	Long et al. (2005)
DBH Purified bovine DA beta hydoxylase	Mouse	Chemicon, Temecula, CA MAB308, USA	1:400	Trigueiros-Cunha et al. (2003), Long et al. (2005), Drescher et al. (2006)

### Image Processing and Analysis

Images were examined using a Leica DM4500 microscope equipped with appropriate band pass fluorescent filters. Images were taken using a Photometrics Coolsnap EZ, 12 bit, 20 MHz monochrome digital camera (Maeger Scientific, Ann Arbor, MI, USA). To analyze data from images, NIS Elements Software (Nikon) was used to identify and determine the density of labeled terminals and cells. For density analysis, the density of labeled TH fibers and cells for each region analyzed was determined bilaterally in three different sections separated by at least 120 μm across animals in normal hearing as well as 3 days and 2 months deaf animals. The results are reported as a scaled ratio per μm^2^. Student’s *t*-test was used to determine significant differences (*p* ≤0.05).

## Results

### Deafness Related Differential Expression of Tyrosine Hydroxylase and Amino Acid Neurotransmitter Related Genes

#### Cochlear Ablation Results in Profound Hearing Loss

Auditory brainstem responses were analyzed before and after cochlear ablation. Average thresholds for normal hearing animals were 30 dB at 4 kHz (SD ± 5.04), 28 (SD ± 2.45) dB at 12 kHz, and 30 dB at 20 kHz (SD ± 4.15). At 3 days and 2 months following cochlear ablation, hearing thresholds were not detectable up to 100 dB (the loudest level tested at each frequency). The results suggest that the surgery was successful in each case assessed.

#### Cochlear Damage Results in Temporal Correlation of Tyrosine Hydroxylase and Inhibitory Neurotransmitter Related Gene Expression in the CN

**FIGURE 1:**
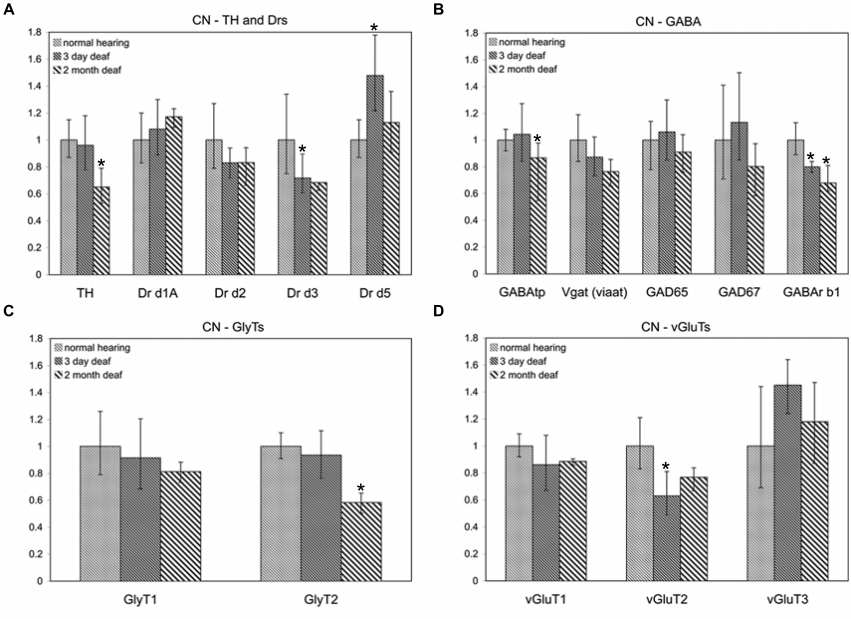
**Dopamine (DA) and amino acid neurotransmitter (AAN) related genes are differentially expressed in the cochlear nucleus (CN).** Changes were assessed in DA related **(A)**, inhibitory **(B,C)** and excitatory **(D)** AAN related gene expression in the CN, 3 days and 2 months following bilateral cochlear ablation. Although TH gene expression levels were not changed at the 3 day time point, expression was significantly decreased by 2 months (35%; **A**). Only two other genes, GABAtp and GLYT2, both inhibitory, had a similar pattern of expression, significant decreases by 2 months, 13 and 42%, respectively. Three days of deafness resulted in DA receptor Drd5, being significantly increased (42%) while Drd3 and VGLUT2 were significantly decreased by 28% **(A)** and 37% **(D),** respectively. Only gene expression of GABArb1 was significantly changed at both time points with 20% (3 days) and 32% (2 months) decreases were observed. Error bars: SD; asterisk: *p* ≤ 0.05.

**FIGURE 2:**
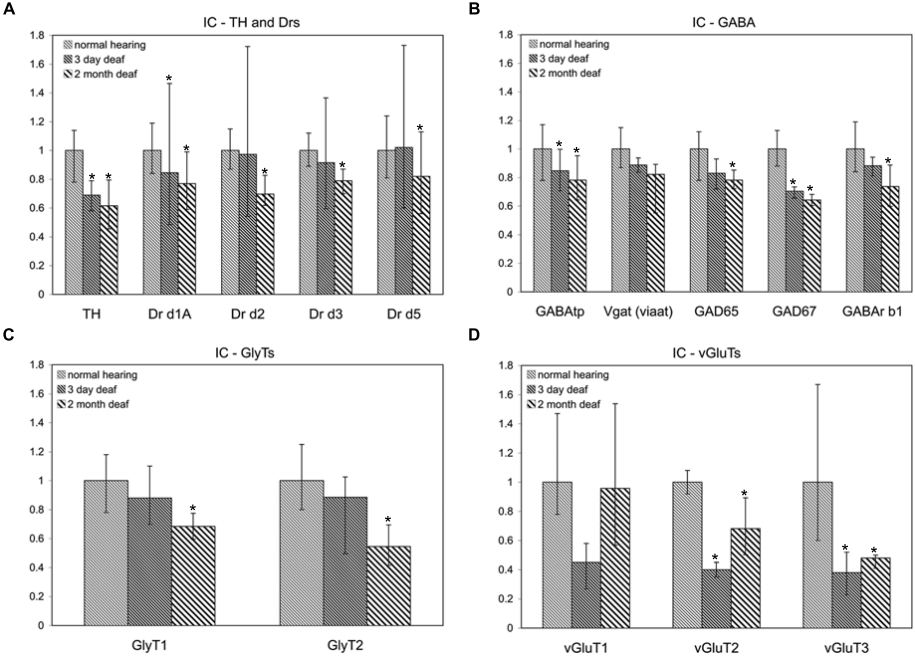
**Dopamine and AAN related genes are differentially expressed in the inferior colliculus (IC).** Changes were assessed in DA related **(A)**, inhibitory **(B,C)**, and excitatory **(D)** AAN related gene expression in the IC 3 days and 2 months following bilateral cochlear ablation. TH expression decreased significantly in the IC at both 3 days (31%) and 2 months (38%). In addition to the DA receptor subunit, Drd1A (15%-3 days; 23%-2 months; **A**), four AAN related genes showed the same pattern of expression as TH: significant decreases at both time points. Of these, two were related to excitatory neurotransmission (VGLUT2: 60%-3 days and 32%-2 months; VGLUT3: 62%-3 days and 52%-2 months) and two related to inhibitory neurotransmission (GABAtp: 15%-3 days and 22%-2 months; GAD67: 29%-3 days and 36%-2 months). Interestingly, seven genes were significantly decreased (Drd2-30%; Drd5:-18%; GLYT1- 31%; GLYT2- 45%; GAD65- 22%; GABArb1- 26%) only at the 2 months time point **(A,C,D)**. Error bars: SD; asterisks: *p* ≤ 0.05.

To determine whether the pattern of differential expression of DA parallels that of AANs related genes, the relative expression level for TH and DA receptors was compared, with that of GABA, glycine, and glutamate related genes by qRT-PCR in the CN and IC (**Figures 1** and **2**) at two time points following cochlear ablation. For the CN TH was significantly decreased (**Figure 1A**) only at the 2 months time point (mean: 0.65, *p* = 0.0032). Of the 14 other neurotransmitter related genes tested, only GABAtp (mean: 0.87, *p* = 0.0110) and GLYT2 (mean: 0.58, *p* = 0.0071) had a similar change in expression (**Figures 1B,C**), decreasing only at the 2 months time point following cochlear ablation. However, four other neurotransmitter related genes also showed significant changes in expression.

Expression of DA receptor Drd3 (mean: 0.72, *p* = 0.00275) was significantly decreased, while Drd5 was significantly increased (mean: 1.48, *p* = 0.00384), 3 days following ablation (**Figure 1A**). The expression of VGLUT2 was significantly decreased (**Figure 1D**) only at the 3 days time point (mean: 0.63, *p* = 0.00011) while GABRB1 was the only gene significantly decreased (**Figure 1B**) at both 3 days (mean: 0.80, *p* = 0.0145) and 2 months (mean: 0.68, *p* = 0.0143) following cochlear damage.

#### Cochlear Damage Results in Temporal Correlation of Tyrosine Hydroxylase and both Excitatory and Inhibitory Neurotransmitter Related Gene Expression in the IC

In accord with previous results (Holt et al., 2005; Tong et al., 2005) significant decreases were observed in the level of TH in the IC both at 3 days (mean: 0.69, *p* = 0.022) and 2 months (mean: 0.62, *p* = 0.014) following cochlear damage (**Figure 2A**). Five of the other genes tested were also significantly decreased at both time points, including DA receptor Drd1A (**Figure 2A**; 3 days mean: 0.85, *p* = 0.007; 2 months mean: 0.77, *p* = 0.028) genes related to inhibitory neurotransmission (**Figure 2B**), GAD67 (3 days mean: 0.71, *p* = 0.0123; 2 months mean: 0.64, *p* = 0.001) and GABAtp (3 days mean: 0.85, *p* = 0.034; 2 months mean: 0.78, *p* = 0.006), as well as those related to excitatory neurotransmission (**Figure 2D**), VGLUT2 (3 days mean: 0.40, *p* = 4.5 × 10^-9^; 2 months mean: 0.68, *p* = 0.029) and VGLUT3 (3 days mean: 0.38, *p* = 0.035; 2 months mean: 0.48, *p* = 0.049). There were additional genes for which expression was decreased only at the 2 months time point. These include DA receptors (**Figure 2A**), Drd2 (mean: 0.70, *p* = 0.010), Drd3 (mean: 0.79, *p* = 0.054), and Drd5 (mean: 0.82, *p* = 0.038) as well as those genes related to inhibitory neurotransmission (**Figures 2B,C**), GABRB1 (mean: 0.74, *p* = 0.017), GAD65 (mean: 0.78, *p* = 0.0004), as well as GLYT1 (mean: 0.69, *p* = 0.011) and GLYT2 (mean: 0.55, *p* = 0.003).

### Differential Localization and Distribution of Tyrosine Hydroxylase and Amino Acid Neurotransmitter Related Proteins Following Hearing Loss

#### Controls

**FIGURE 3:**
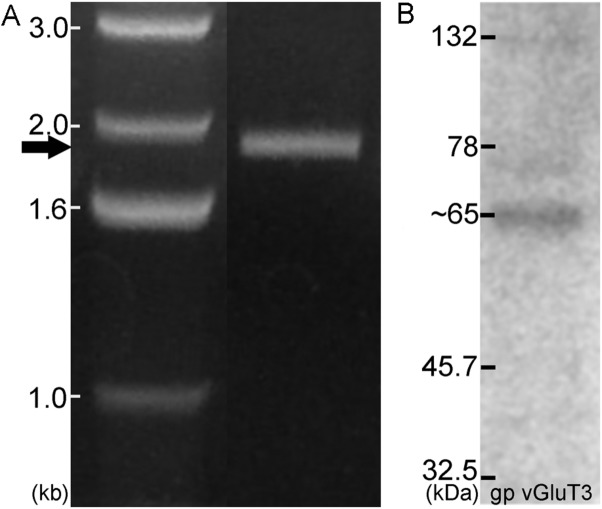
**In the IC VGLUT3 is expressed and produced.** Gene expression for VGLUT3 in the IC was demonstrated using PCR **(A)**, with a predicted band of approximately 1.9 kb. Western blotting for VGLUT3 protein in the IC resulted in a single band ∼65 kDa **(B)**.

Antibodies for each of the proteins assessed have either been previously verified (**Table 1**) or were verified during the course of the current study. The specificity of antibodies for VGLUT1, VGLUT2, and VGLUT3 in the DCN and VCN (Fyk-Kolodziej et al., 2011) as well as VGLUT1 and VGLUT2 in the IC (Altschuler et al., 2008) have been previously verified. In the IC, specificity for VGLUT3 was verified using several methods. First, specific primers were designed for PCR verification of VGLUT3 in the IC. Similar to previous results in the DCN, VCN, and auditory cortex, a band of predicted size (∼1.9 kb) was identified spanning the entire coding region (**Figure 3A**). The PCR products were cloned and sequence verified. No splice variants were identified. This suggests that a single VGLUT3 isoform is present in the IC. In addition, Western blotting (**Figure 3B**) resulted in a single band of the expected size (∼65 kD). Preadsorption of the VGLUT3 antibody with the targeted antigen resulted in great diminution of immunolabeling. Just as in the CN, two different VGLUT3 antibodies were used, The pattern of immunolabeling was the same for both antibodies. Taken together the gene expression and immunohistochemistry data provide evidence that neurons in the IC express and produce VGLUT3.

**FIGURE 4:**
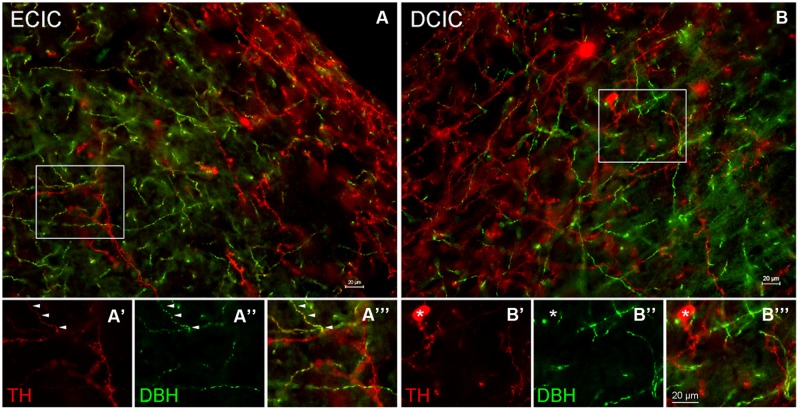
**Co-localization of TH and DBH in the IC reveals three types of labeling.** In the external cortex of the inferior colliculus (ECIC) labeling for TH (red) and TH + DBH (yellow) is observed throughout the nucleus **(A)**. The majority of fibers and terminals in the deeper layers of the ECIC were co-localized for DBH and TH (e.g., arrows) and occasional labeling for fibers containing only TH were observed **(A’–A”’)**. In the more superficial layers of the ECIC labeling for TH is predominant **(A)**. In the dorsal cortex of the IC (DCIC) labeling for TH and DBH was observed throughout the subdivision **(B)**. There were somata, fibers and terminals that labeled only for TH (red), some fibers that labeled for both TH and DBH (yellow) and a population of fibers that appeared to label only for DBH **(B’–B”’)**. Asterisk: somata labeled only for TH Scale bar: 20 μm.

In the CN the first step was to determine whether dopaminergic somata and terminals are found in the DCN and AVCN. The rate-limiting enzyme, TH, was used as a marker for DA. The catecholaminergic transmitters DA, nor-adrenaline, and adrenaline are synthesized through a common pathway. There are several enzymes that are part of the biosynthetic machinery including: phyenylalanine hydroxylase, TH, aromatic Dopa decarboxylase (ADC), dopamine β hydroxylase (DBH), pteridine reductase, and PNMT. When compared to DBH and PNMT, labeling of a neuron for TH only indicates DA production, while labeling of a neuron for TH and DBH indicates adrenaline or noradrenaline, and a neuron that is positive for TH, DBH, and PNMT would be expected to produce adrenaline. In the AVCN, while there were many fibers labeled only for TH, there were fibers that co-labeled for both TH and DBH (data not shown). In addition, as previously reported in the IC (Tong et al., 2005), the majority of TH positive somata are DBH negative (**Figure 4**). However, three different populations of fibers were observed. In both the DCIC and ECIC, fibers labeled for TH only were predominant in the most dorsal layers (**Figures 4A,B**). Fibers co-labeled for both TH and DBH were primarily found in the ventral ECIC (**Figures 4A’–A”’**). The majority of fibers in the more ventral regions of the DCIC labeled for DBH only (**Figures 4B’–B”’**).

#### Localization of Tyrosine Hydroxylase in the CN and IC Following Cochlear Damage

##### AVCN

**FIGURE 5:**
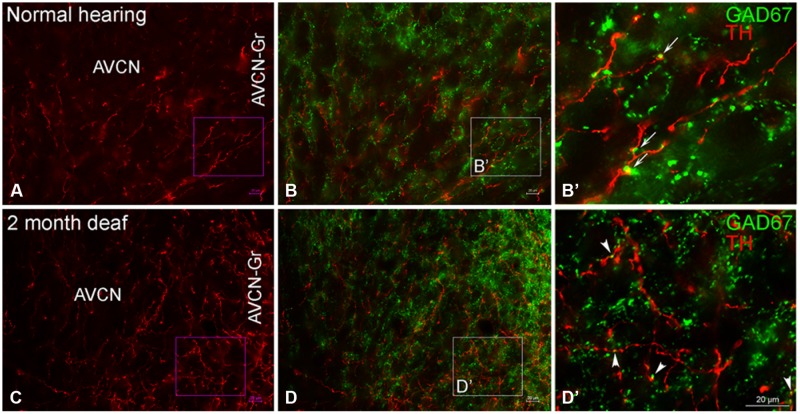
**In the AVCN the majority of GAD67 (green) has a distribution that is distinct from TH (red).** While GAD67 is primarily localized to organized axosomatic GABAergic terminals **(B,B’)**, TH on the other hand, appears to be localized both to dendrites and axon terminals **(A,C)**. Two months after deafness was induced, GAD67 labeling is increased in the granule cell region and the distribution to larger terminals forming axosomatic rings is disrupted **(D,D’)**. Arrows **(B’)** and arrowheads **(D’)** indicate representative areas of potential GAD67 and TH contact. Scale bar: 20 μm.

In normal hearing animals modest labeling for TH was distributed throughout the AVCN, including the granule cell region (**Figure 5A**, red). Labeling for TH was observed primarily in en passant fibers throughout the nucleus. Two months following cochlear ablation, the overall density of TH labeling was decreased by 17% in the AVCN (**Figure 5C**).

##### DCN

**FIGURE 6:**
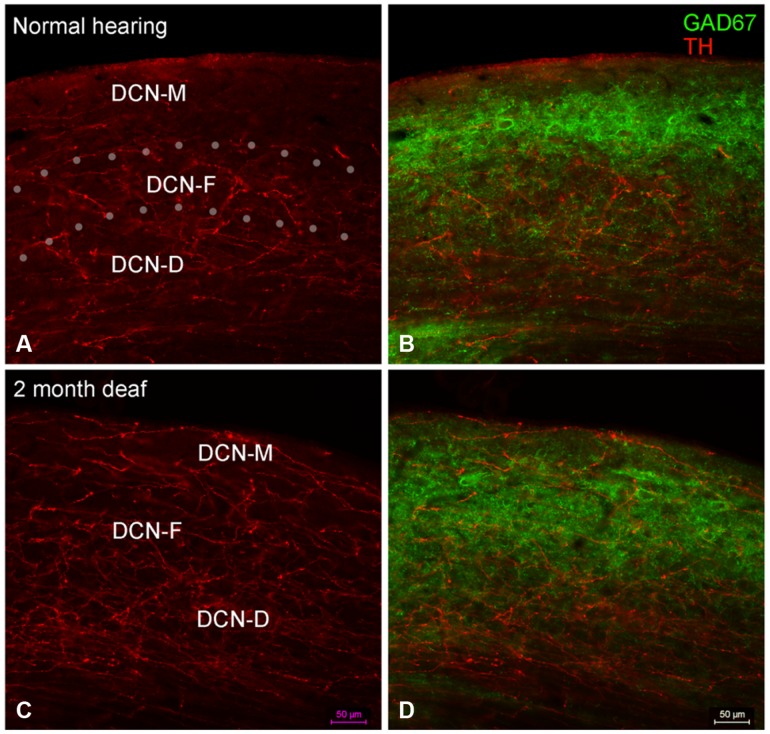
**In the DCN the GAD67 labeling (green) is distinct from TH (red) with sparse labeling for TH fibers in DCN-M **(A,C)** and GAD67 (B,D) primarily localized to terminals with distinct labeling in ventral DCN-M and DCN-F.** Following deafness GAD67 labeling increases in DCN-M and DCN-D **(D)** while for TH labeling there is an increase in DCN-M boutons and a decrease in the neuropil **(C)**. Fine GAD67 labeled boutons do not extend to the deepest layers of DCN-D 2 months after deafening. Dotted lines **(A)** indicate approximate boundaries between DCN-M, DCN-F, and DCN-D. Scale bar: 50 μm.

Labeling for TH is also observed throughout the DCN (**Figure 6A**) with labeling for fibers most sparse in the molecular layer (DCN-M). Following 2 months of deafness the general density of TH labeling changes, with less labeling in the neuropil and more labeling in boutons and fibers of passage (**Figure 6C**). The change in density of TH labeling is fairly uniform, observed in the superficial DCN-M, the fusiform layer (DCN-F) as well as the deep layer (DCN-D) of the DCN, (**Figure 6C**).

##### Inferior Colliculus

Several studies have reported the distribution of TH in the IC (Holt et al., 2005; Tong et al., 2005; Hormigo et al., 2012). In normal hearing rats, the most robust labeling for TH positive somata is in the dorsal portion of the caudal DCIC, while rich immunolabeling for fibers and terminals is found throughout the IC. Following hearing loss differential labeling of TH has been reported within subdivisions of the IC at different times following cochlear damage. (Holt et al., 2005; Tong et al., 2005). The results of the present study are consistent with these previous results found after 3 months of induced deafness. In the current study TH labeling is decreased in the IC at the 2 months time point (data not shown).

### Co-Localization of TH and Amino Acid Neurotransmitters in the CN Following Cochlear Damage

#### Co-immunolabeling for TH and GAD67 in the Cochlear Nucleus Reveals a Largely Complementary Distribution

##### AVCN

While TH labeling (red) is localized to both dendrites and axon terminals, GAD67 (green) is prominent within GABAergic terminals (**Figures 5B,B’**) with axosomatic profiles surrounding (a ring of greater than ten GAD67 profiles) immunonegative somata. 2 months following induced deafness the density of GAD67 labeling is not changed (normal: 1.15 ±0.07; deaf: 1.21 ± 0.06). However, axosomatic labeling, as demonstrated by organized perisomatic rings (**Figures 5B,B’, D,D’**) decreases (normal: 19 ± 1.7; deaf: 9 ± 2.8), replaced with a more disorganized, diffuse axodendritic labeling within the neuropil. In addition, more robust GAD67 labeling is observed in the granule cell region (**Figure 5D**) with labeling in this region increasing by 50%. Although very little co-localization of TH and GAD67 was observed in normal hearing animals (**Figures 5B,B’**), there was a close association of TH and GAD67, in both the normal and deaf groups (**Figures 5B,B’, D,D’**). Following 2 months of induced deafness GAD67 is localized to smaller immuno-positive profiles.

##### DCN

In normal hearing animals, perisomatic, and somatic labeling for GAD67 was observed in all layers of the DCN (**Figure 6B**), with labeling in the dorsal most portion of the DCN-M being most sparse and labeling in more ventral DCN-M and the DCN-F layer being most dense. In the DCN-D, the heaviest GAD67 labeling is localized to the most ventral portion of the region. Following deafness, GAD67 labeling in the DCN-M was enhanced. However, in the DCN-F labeling was less intense and more diffuse. In the DCN-D labeling was diminished. Very few sites of possible TH and GAD67 interaction were observed under normal hearing conditions (**Figure 6B**) or following cochlear damage (**Figure 6D**).

#### Immunolabeling for TH and Glycine Transporter 2 (GLYT2) in the Cochlear Nucleus Appears Less Robust Following Cochlear Damage

##### AVCN

**FIGURE 7:**
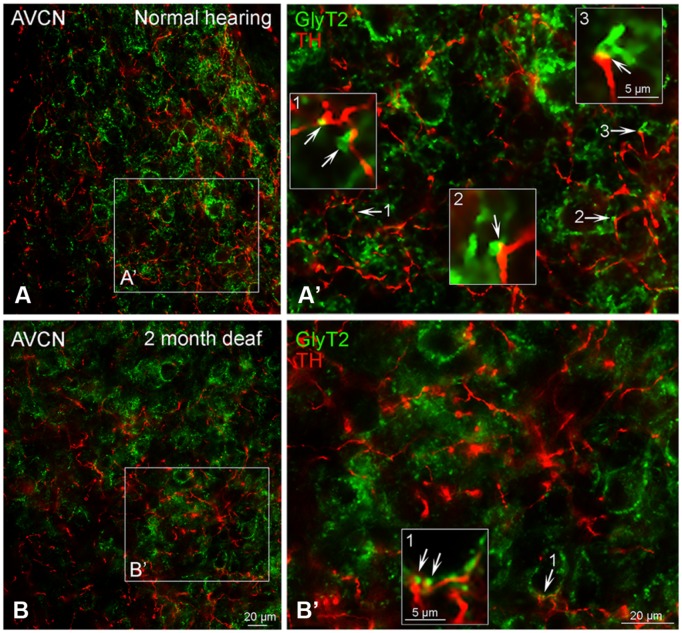
**In the AVCN the distribution of GLYT2 (green) overlaps with TH (red).** Labeling for GLYT2 is localized to glycinergic terminals with TH labeling primarily localized to complimentary terminals and dendrites **(A,B)** with a few areas of co-localization (yellow). Following deafness GLYT2 labeling is decreased. Arrows indicate closely associated puncta. **(A’)** – an enlargement of square in **(A)**. **(B’)** – an enlargement of box in **(B)**. Numbers and arrow in **(A’)** and **(B’)** indicated enlarged regions in **(A’,B’)** with arrows showing co-localization (yellow) and close apposition between GLYT2 and TH. Scale bar: 20 μm.

In normal hearing subjects, GLYT2 labeled terminals were distributed throughout the AVCN and localized to both large (>1.50 μm) and small (0.4–1.5 μm) axosomatic terminals (**Figures 7A,A’**). Many of the TH and GLYT2 positive terminals appeared to be in close apposition (**Figures 1–3** and **7A**’). Hearing loss shifted labeling of GLYT2 axon terminals primarily to small terminals (**Figures 7B,B’**). Also fewer perisomatic GLYT2 labeled terminals were apparent. Nonetheless, a robust interaction (possible contact) persisted between TH positive fibers and the remaining GLYT2 perisomatic terminals (**Figures 1** and **7B’**).

##### DCN

**FIGURE 8:**
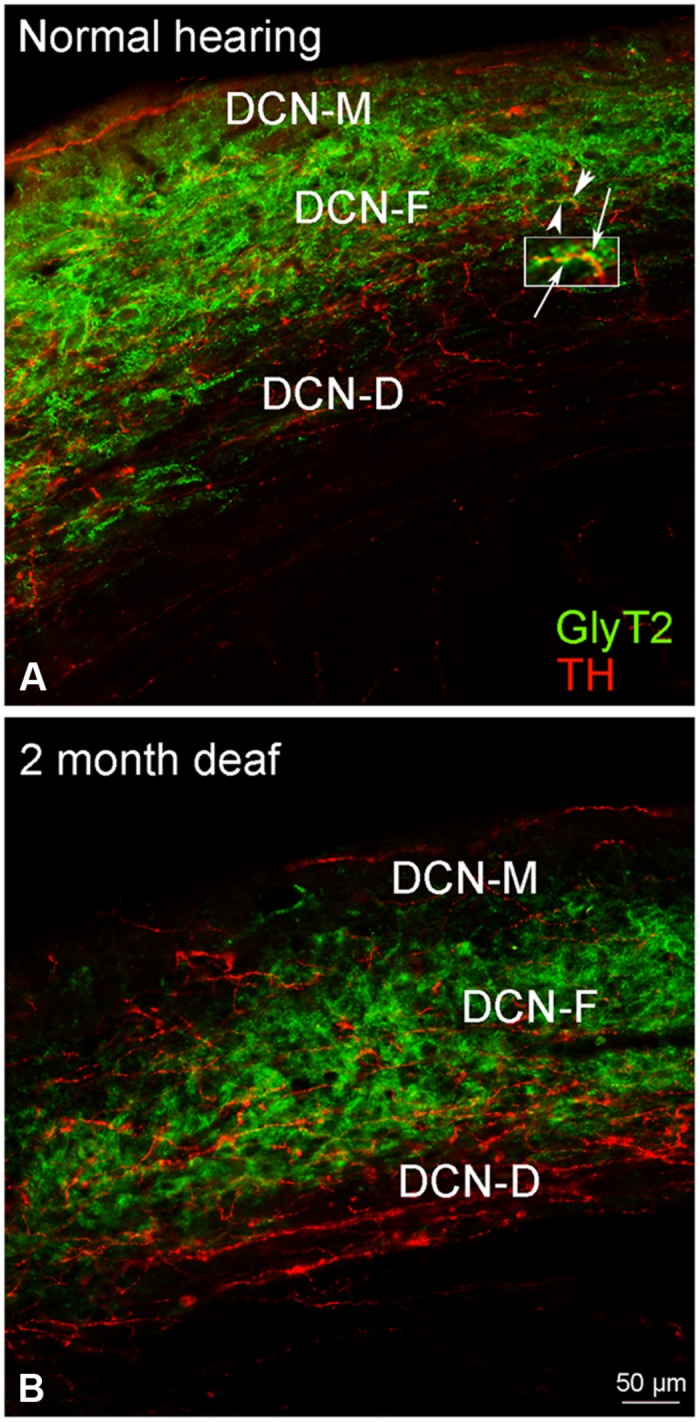
**In the DCN, GLYT2 labeling is robust throughout most of the DCN.** While both GLYT2 (green) and TH (red) labeling is localized to dendrites and terminals **(A,B)**, the labeling is primarily complimentary. Following deafness GLYT2 labeling is dramatically decreased in each layer of the DCN, especially in DCN-M and DCN-D. Arrowheads indicate regions of TH and GLYT2 interaction enlarged at arrows. Scale bar: 50 μm.

Although immunolabeling for GLYT2 was distributed throughout all three layers of the DCN (**Figure 8A**), the bulk of the labeling was in the molecular and fusiform layers. Cochlear damage resulted in a decrease in GLYT2 labeling throughout the DCN, but with a particularly dramatic decrease in GLYT2 observed in DCN-M and DCN-D (**Figure 8B**). Co-localization and/or possible contact between TH and GLYT2 was noted (**Figure 8A**) and did not seem affected by deafness.

#### Co-Immunolabeling for TH and Vesicular Glutamate Transporter 1 (VGLUT1) in the Cochlear Nucleus Demonstrates a Possible Relationship

##### AVCN

**FIGURE 9:**
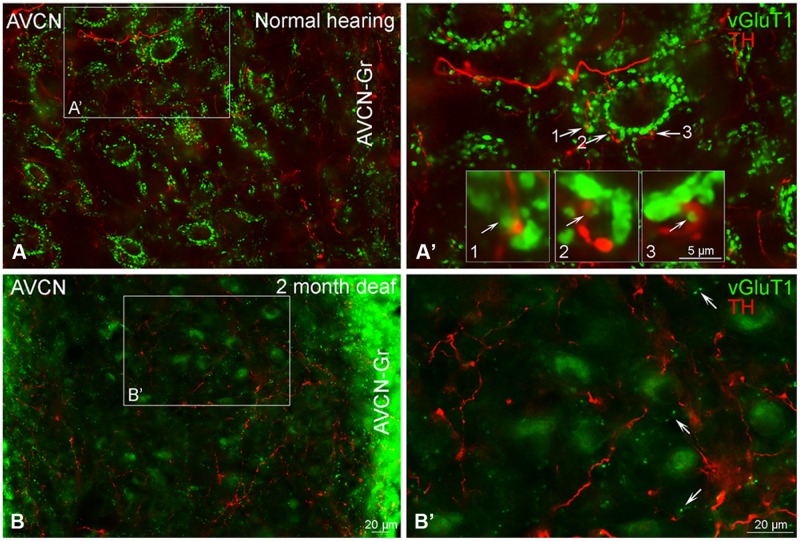
**Vesicular Glutamate Transporter 1 (green) is found in the anteroventral cochlear nucleus (AVCN) with a distribution largely distinct from TH (red).** In normal hearing animals labeling for VGLUT1 is primarily localized to large glutamatergic terminals **(A,A’)** forming axosomatic ring structures throughout the nucleus **(A,B)**. While VGLUT1 positive terminals are in close contact with TH positive en passant swellings (**A’** 1–3) little to no co-localization was observed. Following deafness, labeling for VGLUT1 becomes visible within cell bodies and smaller terminals **(B,B’)**. Scale bar – 5 μm in boxes labeled 1, 2, and 3. Scale bar: 20 μm **(A,A’,B,B’)**.

Just as reported previously (Fyk-Kolodziej et al., 2011), when there is no substantial cochlear damage, VGLUT1 labeling is localized to axosomatic and axodendritic terminals throughout the AVCN (**Figures 9A,A’**). Severe cochlear damage results in a dramatic shift in VGLUT1 labeling from terminals to somata in the core of the AVCN with a pronounced increase in VGLUT1 labeling in the granule cell domain (**Figures 9B,B’**). While no co-localization of TH and VGLUT1 was observed under normal hearing conditions, there appeared to be possible contact between VGLUT1 and TH positive boutons (**Figures 1–3** and **9A’**). Following deafness this close association of TH and VGLUT1 was severely diminished (**Figure 9B’**), with little putative contact observed between VGLUT1 somata and TH positive terminals.

##### DCN

**FIGURE 10:**
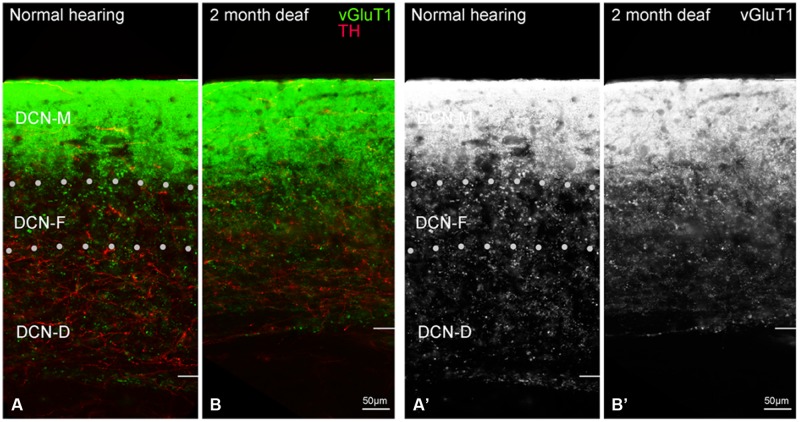
**In the dorsal cochlear nucleus (DCN) the distribution of VGLUT1 (green) is distinct from TH (red).** In normal hearing animals VGLUT1 is primarily localized to glutamatergic terminals that rarely co-localize with TH labeled terminals **(A)**. Following deafness VGLUT1 labeling is more diffuse and less punctate in DCN-F **(B,B’)**. In panels **(A’,B’)** only the green channel (VGLUT1) is depicted Scale bar: 50 μm.

Labeling of VGLUT1 has been reported in the DCN (Zhou et al., 2007; Fyk-Kolodziej et al., 2011). Labeling for VGLUT1 is found within terminals in all three layers of the DCN (**Figures 10A,A’**), with the heaviest labeling in the molecular layer (DCN-M). While synaptic contact between and/or co-localization of VGLUT1 and TH in the molecular layer may be expected, the density of VGLUT1 labeling precluded us obtaining clear evidence of such interactions. Deafness resulted in diffuse VGLUT1 labeling in DCN-F and DCN-D with few VGLUT1 TH points of contact (**Figures 10B,B’**).

### Co-localization of TH and Amino Acid Neurotransmitters in the IC

#### Co-Immunolabeling for TH and GAD67 in the Inferior Colliculus Reveals a Largely Complementary Distribution

**FIGURE 11:**
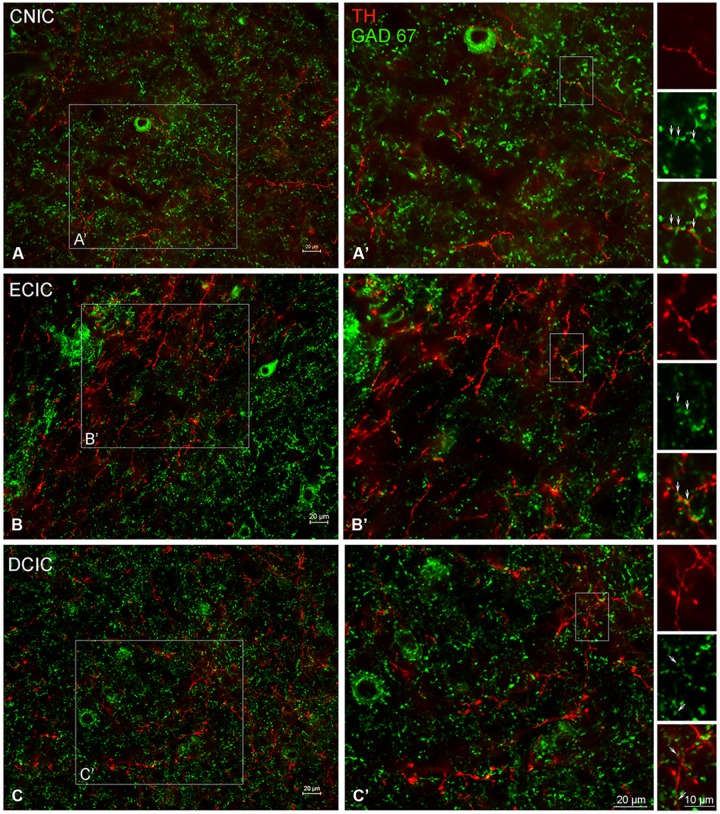
**In the IC GAD67 (green) is distributed throughout each subdivision and is closely associated with TH labeled (red) en passant terminals.** Terminals labeled for GAD67 in the central nucleus (CNIC), external cortex (ECIC), and dorsal cortex (DCIC) of the IC **(A–C)** are in close contact with TH labeled terminals **(A’,B’)**. Arrows indicate TH labeled spines studded with GAD67 labeled puncta. Scale bar: 10 μm in boxes containing arrows. Scale bar: 20 μm **(A,A’–C,C’)**.

The central nucleus (CNIC), external cortex (ECIC), and dorsal cortex (DCIC) of the IC were examined for co-localization of TH and GAD67 (**Figure 11**). Labeling for GAD67 was observed in somata and axon terminals throughout the CNIC (**Figures 11A,A’**). Interspersed amongst the GAD67 labeled somata and axon terminals, TH labeled fibers were found to be in very close proximity to GAD67 terminals (**Figure 11A**’ – enlargements). This same pattern of GAD67 labeling was observed in the DCIC (**Figures 11C,C’**). However, in the ECIC, while GAD67 labeling was present through the region, layer two had noticeably less labeling (**Figures 11B,B’**). Although sparse, occasional co-localization of GAD67 and TH was observed in en passant swellings in the ECIC and the DCIC (see enlargements in **Figures 11B’,C’**).

#### Immunolabeling for TH and GLYT2 in the Inferior Colliculus is Co-Localized

**FIGURE 12:**
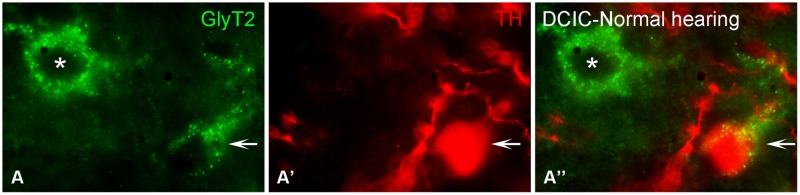
**In the IC GLYT2 (green) and TH (red) are co-localized.** In the DCIC **(A–A”)** double labeling for TH and GLYT2 was primarily observed in somata and dendrites. Arrows indicate somatic labeling of GLYT2 and TH. The asterisk indicates a neuron in the DCIC labeled for GLYT2 only **(A)**. DCIC – dorsal cortex of the IC; Scale bar: 5 μm.

Somata and terminals immunopositive for GLYT2 were distributed throughout the CNIC, ECIC, and DCIC. Interestingly, the CNIC contained the most sparse labeling of GLYT2 somata and the greatest density of GLYT2 terminals, which was localized to the ventral CNIC (Data not shown). Co-localization of GLYT2 with TH within somata was observed in both the DCIC (**Figures 12A–A”**) and the ECIC (Data not shown).

#### Terminals Immunolabeled for TH and VGLUT1 are Closely Associated in the IC

**FIGURE 13:**
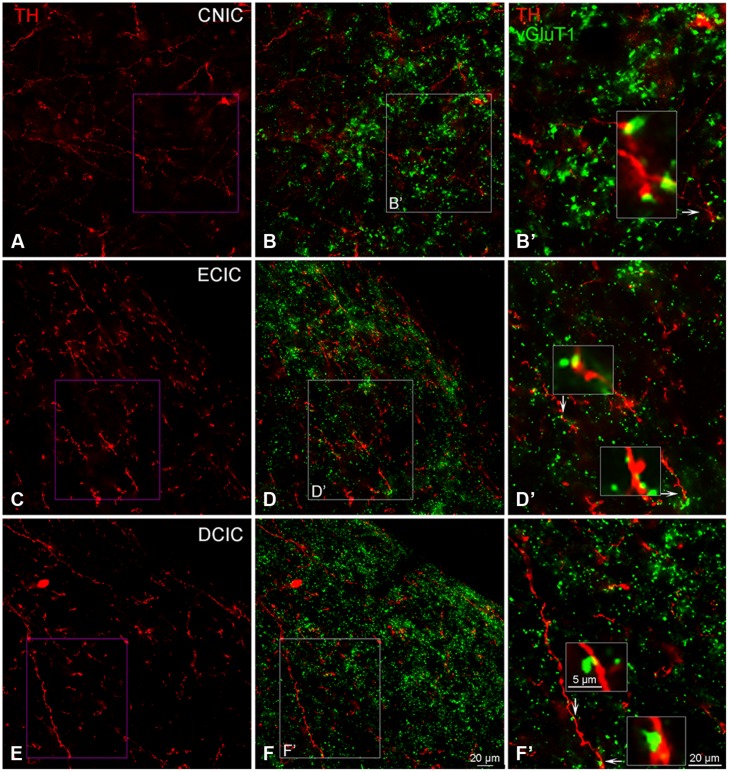
**In the IC VGLUT1 (green) is distributed throughout each subdivision and is closely associated with TH labeled (red) terminals.** Terminals labeled for TH in the CNIC, ECIC, and DCIC **(A,C,E)** appear to be in close contact with VGLUT1 labeled terminals **(B,B’,D,D’,F,F’)**. Arrows indicate terminals enlarged within boxes in **(B’,D’)** and **(F’)**. Boxes in **(B,D,F)** are enlarged in **(B’,D’,F’)**; Scale bar: 5 μm for boxes in **(B’,D’,F’)**. Scale bar: 20 μm **(A,B,B’,C,D, D’,E,F,F’)**.

The distribution of VGLUT1 has previously been reported in the IC (Altschuler et al., 2008). In the IC, labeling for VGLUT1 was restricted to axon terminals (**Figure 13**). Within each subdivision of the IC, VGLUT1, and TH were in close apposition (**Figures 13B,D,F**), with VGLUT1 often present along TH labeled *en passant* fibers (**Figures 13B’,D’,F’**).

#### Vesicular Glutamate Transporter 3 (VGLUT3) is Distributed Throughout the Inferior Colliculus and Co-Localized with TH

**FIGURE 14:**
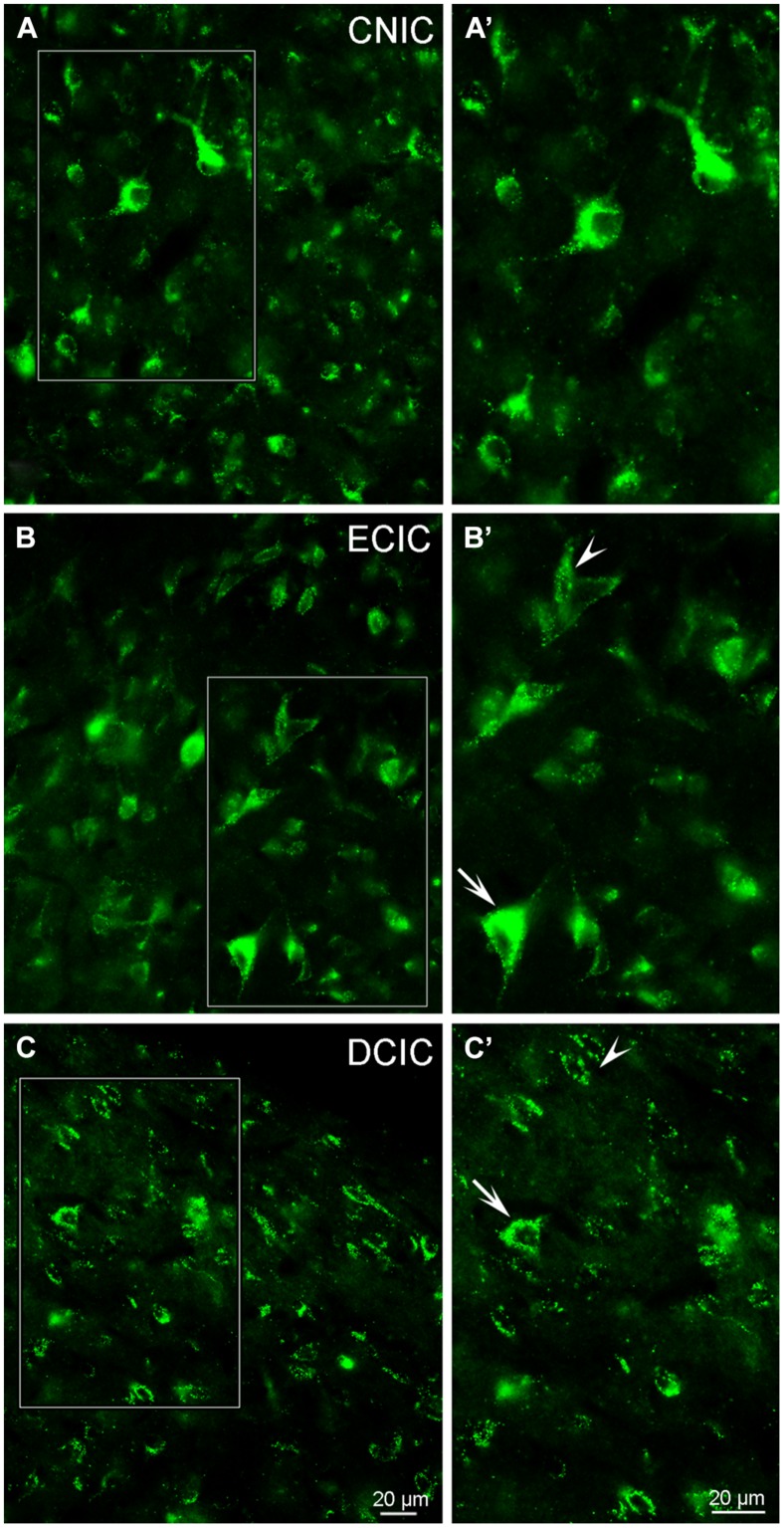
**In the IC VGLUT3 is distributed throughout each subdivision.** Although some labeling is observed in terminals, the majority of labeling for VGLUT3 is contained within somata and dendrites **(A–C)**. In the CNIC large and small multipolar neurons with round somata and proximal dendrites are labeled for VGLUT3 **(A,A’)**. While prominent somatic labeling for VGLUT3 is found in the ECIC **(B,B’)** and DCIC **(C,C’)** localized to small elongated neurons with little to no labeling of proximal dendrites (arrowheads) as well as within larger neurons, including proximal dendrites (arrows). CNIC – central nucleus of the IC; ECIC – external cortex of the IC; DCIC – dorsal cortex of the IC; Scale bars: 20 μm.

**FIGURE 15:**
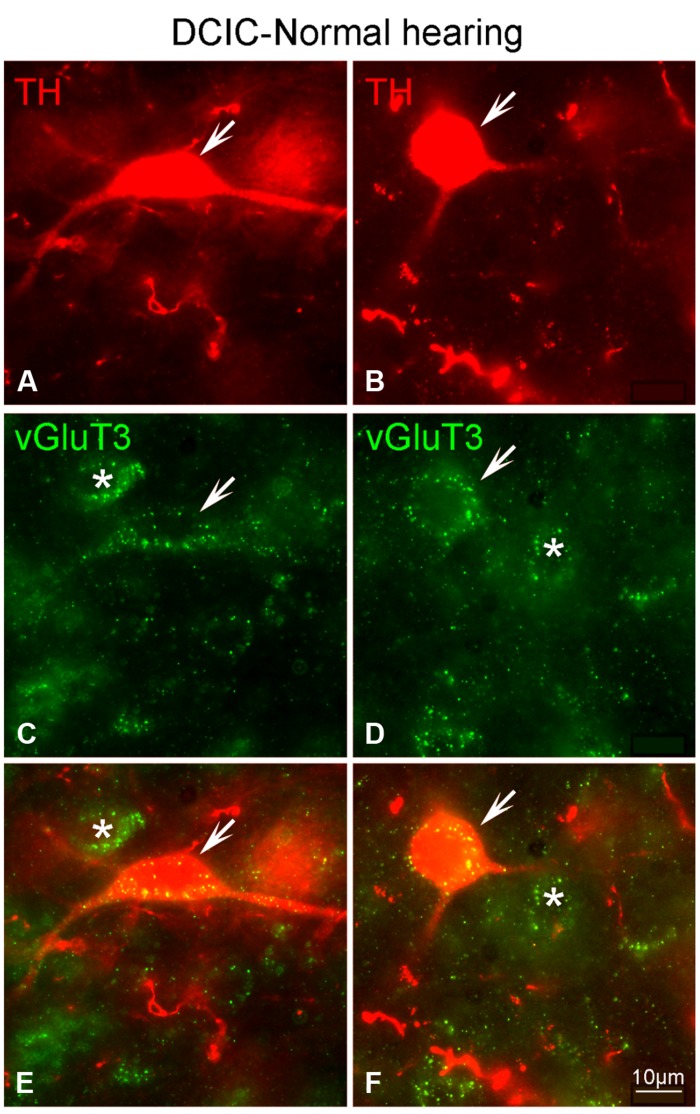
**In the DCIC VGLUT3 (green) and TH (red) are co-localized in somata and dendrites (A–F).** In the DCIC double labeling for VGLUT3 and TH was primarily observed in somata and dendrites. Arrows indicate somatic and dendritic labeling for VGLUT3 and TH. The asterisks indicate neurons in the DCIC labeled only for VGLUT3. DCIC – dorsal cortex of the IC; Scale bars: 10 μm.

Although labeling for VGLUT3 was observed in fine terminals within the IC, the most robust labeling was punctate and primarily observed within large and small somata (**Figure 14**). While VGLUT3 labeling was clearly localized to both somata and dendrites in the CNIC (**Figures 14A,A’**) and ECIC (**Figures 14B,B’**), in the DCIC VGLUT3 labeling appeared to be predominantly confined to somata (**Figures 14C,C’**) and was less evident in dendrites. Co-immunolabeling of VGLUT3 and TH was largely observed in the DCIC (**Figure 15**). Approximately 25% of the VGLUT3 immunolabeled somata in the DCIC co-contained TH, primarily in stellate and disk shaped neurons (**Figures 15A–F**).

## Discussion

In the current study, the distribution of TH in the CN has been detailed and previous work demonstrating the presences of TH in the IC has been substantiated (Holt et al., 2005; Tong et al., 2005; Hormigo et al., 2012; Gittelman et al., 2013). Described for both the CN and the IC, are deafness (cochlear ablation) induced temporal changes in gene expression for TH, DA receptors and genes related to AANs (**Tables 2** and **3**). The current study adds to growing evidence supporting the presence of endogenous DA and dopamine receptors reported in the CN and IC (Wamsley et al., 1989; Weiner et al., 1991; Holt et al., 2005; Tong et al., 2005; Bender et al., 2010, 2012; Hormigo et al., 2012; Maison et al., 2012; Gittelman et al., 2013; de Oliveira et al., 2014; Muthuraju et al., 2014). As a neuromodulator, DA in the CN affects T-type Ca^+2^ channels at the axon initial segment (Bender et al., 2012). Roles for DA in the IC remain to be determined, but several studies suggest a role for DA in the response to aversive stimuli. In the current study, cochlear damage results in significant changes in gene expression for TH in both the CN and the IC. The close association of TH with GAD67, GLYT2, VGLUT1 as well as VGLUT3 places DA in a position to influence the balance of excitation and inhibition in the CN and IC, prior to and after hearing loss.

**Table 2:** Gene expression and immunocytochemistry comparison in the cochlear nucleus (CN).

	Cochlear Nucleus						
	PCR		ICC				
Genes	3 days	2 months	2 months	2 months	2 months	2 months	2 months
Subdivision	CN	CN	AVCN-C	AVCN-Gr	DCN-M	DCN2-F	DCN-D
VGLUT1	–	–	**↓**	**↑**	**–**	**–**	**↓**
VGLUT2	**↓**	–					
VGLUT3	–	–					
GAD65	–	–					
GAD67	–	–	–	**↑**	**↑**	**↓**	**↓**
GLYT1	–	–					
GLYT2	–	**↓**	–	–	**↓**	**↓**	**↓**
TH	–	**↓**	**↓**	**↑**	**↓**	**↓**	**↓**
GABAtp	–	**↓**					
GABArbl	**↓**	**↓**					
VGAT	–	–					
DrdlA	–	–					
Drd2	–	–					
Drd3	**↓**	–					
Drd5	**↑**	–					

*(a) Up arrow – Increased levels. (b) Down arrow – Decreased levels. (c) Dashes – No Change. (d) Empty – Not assessed.*

**Table 3:** Gene expression and immunocytochemistry summary in the inferior colliculus (IC).

	IC				
	PCR		ICC		
Genes	3 days	2 months	Normal hearing	Normal hearing	Normal Hearing
Subdivision/Genes	IC	IC	CNIC	DCIC	ECIC
VGLUT1			+	+	+
VGLUT2	**↓**	**↓**			
VGLUT3	**↓**	**↓**	+	+	+
GAD65		**↓**			
GAD67	**↓**	**↓**	+	+	+
GLYT1		**↓**			
GLYT2		**↓**	+	+	+
TH	**↓**	**↓**			
GABAtp	**↓**	**↓**			
GABArbl		**↓**			
VGAT					
DrdlA	**↓**	**↓**			
Drd2		**↓**			
Drd3		**↓**			
Drd5		**↓**			

### Genes Related to Dopaminergic and Amino Acid Neurotransmitter (AAN) Effects in the CN and IC are Differentially Expressed Following Deafness

The deafness related changes in gene expression observed in the CN showed a different magnitude and time course of change when compared to changes observed in the IC (summarized in **Tables 1** and **2**). For TH deafness related decreases in gene expression in the CN were only observed at the 2-months time point. Of the AAN related genes, only two genes showed deafness related changes with a similar time course as TH, GLYT2, and GABAtp. Glycine and GABA are the major inhibitory neurotransmitters in the CN. The GABA transporter protein, GABAtp removes GABA from the synaptic cleft. Likewise, the glycine transporter, GLYT2, functions to take up released glycine. The distribution of GLYT2 has been described in the CN and IC (Zafra et al., 1995; Friauf et al., 1999) If levels of these transporters decrease, as in the current study, then GABA and glycine may remain active in the synapse for longer periods resulting in compensatory decreases in production presynaptically. Ototoxicity resulting in profound hearing loss diminishes glycine labeling in the CN (Buras et al., 2006).

In agreement with previous studies (Holt et al., 2005; Tong et al., 2005), following deafening, decreases in TH expression within the IC were evident early and sustained. This change in TH expression correlated with sustained changes in gene expression for GABAergic markers (GABAtp and GAD67), and glutamatergic markers (VGLUT2 and VGLUT3). Interestingly, unlike the CN, in the IC there were no acute changes in expression that were not sustained at the later time point (**Tables 1** and **2**). Also, DA receptor expression at the 3 days deaf time point had the largest SD when compared to other gene expression changes tested in the IC. Increased variability in DA receptor expression observed after 3 days of deafness may reflect a period of instability in DA receptor expression during the time immediately after cochlear ablation. Perhaps this initial period of hearing loss creates flux/plasticity and diminished DA levels as a new homeostatic set point is established. Together, these correlated changes in gene expression suggest that changes in DA levels may modulate neurotransmission in both the CN and IC following deafness.

### TH and AAN Related Proteins in the CN are Closely Associated

In the CN, glycine and GABA positive terminals are in close contact with TH positive terminals and dendrites, providing an opportunity for DA to influence the release or the efficacy of glycine and GABA that has been released. Immunocytochemistry for TH and VGLUT1 in the AVCN reveal labeling primarily in puncta for VGLUT1 and in puncta and dendrites for TH. Following deafness the localization of VGLUT1 labeling shifts from labeling of puncta to labeling of both puncta and somata. However, localization of TH labeling does not appear to change following deafness, but the labeling (intensity and number of elements) is decreased. In the DCN heavy labeling for VGLUT1 is found in DCN-M with sparse labeling in DCN-F and DCN-D of the DCN. Labeling for TH in the DCN is found in each layer. Following deafness, VGLUT1 labeling is decreased in layer 2 and layer 3. There are also deafness related changes in TH labeling with increased labeling in DCN-M and decreased labeling in DCN-F and DCN-D. Immunocytochemistry for GAD67 shows labeled puncta, primarily associated with somata, throughout the AVCN, with prominent labeling of terminals in layer two of the DCN. Deafness results in an increase in GAD67 labeling in the granule cell region and labeling is no longer associated primarily with somata. In the DCN GAD67 labeling is increased in DCN-M and DCN-F. Immunolabeling for GLYT2 in both the AVCN and DCN is localized to terminals with labeling in the AVCN associated with somata and labeling in the DCN most prominent in DCN-F. After 2 months of deafness, labeling decreases throughout the AVCN and in DCN-M and DCN-F. In the CN markers for both DA and AAN related genes decrease and there are changes in the localization as well as decreases in the number and intensity of immunolabeled neuronal elements as a consequence of deafness. While TH appears to be closely associated with AANs in the CN, synapsing with TH labeled processes, there appears to be very little if any co-localization. Although there are deafness related changes in synaptic organization, the opportunity for DA release to impact CN neurons still exists, given the close proximity of TH labeled varicosities. The impact of diminished DA release in the CN could be many fold and would depend not only upon other neurotransmitters at the synapse, but also the type of DA receptor present. In the CN, recent studies suggest D3 mediated modulation of T-type calcium channels can shape spontaneous neuronal activity (Bender et al., 2012). This correlates well with the current study in which DRD3 gene expression is significantly decreased after hearing loss. Although the gene expression studies included the entire CN, these results provide clues as to the cell type that may be contributing to changes since cartwheel cells produce calcium channels at the axon initial segment that are modulated by DA (Bender et al., 2012) and have reduced glycine levels following deafness (Buras et al., 2006).

### While IC Somata Co-Localize TH and either GLYT2 or VGLUT3, Terminals in the IC are Immuno-Positive for either TH or AAN Related Proteins, which are in Close Proximity to One Another

Previously, DA positive terminals and receptors have been reported throughout the auditory pathway, including the IC (Wamsley et al., 1989; Weiner et al., 1991; Kitahama et al., 1996; Tong et al., 2005; Goodson et al., 2009; Bender et al., 2010; Kubikova and Kostal, 2010; Kubikova et al., 2010; Maison et al., 2012). DA in the IC has been shown to have functional relevance. Introduction of DA agonists and D2 receptor antagonists into the IC results in modulation of the acoustic startle reflex (Satake et al., 2012), noted for use in human studies and animal models of psychiatric illness (Swerdlow et al., 2008). Pre-pulse inhibition of the acoustic startle reflex, has more recently been adapted (gap inhibition of the acoustic startle reflex) and used in the study of tinnitus (Turner et al., 2006; Holt et al., 2010; Fournier and Hebert, 2012). In the DCIC, ECIC and the CNIC of the IC immunolabeling for vesicular glutamate transporter 3 (VGLUT3) as well as TH is localized to somata, dendrites and puncta. Following deafness TH labeling decreases throughout the IC, but VGLUT3 immunolabeling shows no change. The labeling for TH and VGLUT3 is co-localized throughout the IC with the most distinct labeling in the DCIC. Labeling for GLYT2 is observed in somata, dendrites and puncta throughout the IC and also co-localizes with TH. Therefore, with TH produced and presumably released from neurons that also produce either glutamate or glycine, TH is positioned to influence the effect of the neurotransmitter released from these neurons. In addition, many TH positive processes are in close contact with AAN containing processes and could, therefore, impact excitability depending upon the type of postsynaptic DA receptors present.

## Conclusion

Deafness results in sustained changes in gene expression for AAN related genes in the CN and the IC with the IC exhibiting more sustained changes. Several of these genes demonstrated decreased expression on a timescale similar to that of TH. Studies demonstrating TH positive fibers in the CN and IC that do not label for DBH are suggestive of DA release in these regions. This idea is further supported by *in situ* studies showing the localization of DA receptor related genes in the CN and IC. The current study adds to the suggestion of endogenous DA in auditory pathways by demonstrating deafness related changes in both D1 and D2 type receptors. DA may therefore play a role in modulating neurotransmitter release in both the CN and IC and/or efficacy of glutamatergic and glycinergic projections from IC neurons. These studies lay the groundwork for future studies on the effects of DA modulation on the release of AANs in the CN and IC.

## Conflict of Interest Statement

The Reviewer Douglas Vetter declares that, despite being affiliated to the same institution as Associate Editor Paul J. May, the review process was handled objectively. The Associate Editor Paul J. May declares that, despite being affiliated to the same institution as Reviewer Douglas Vetter, the review process was handled objectively. The authors declare that the research was conducted in the absence of any commercial or financial relationships that could be construed as a potential conflict of interest.
